# Exploring Clinical and Biological Features of Premature Births among Pregnant Women with SARS-CoV-2 Infection during the Pregnancy Period

**DOI:** 10.3390/jpm12111871

**Published:** 2022-11-08

**Authors:** Ingrid Hrubaru, Andrei Motoc, Felix Bratosin, Ovidiu Rosca, Roxana Folescu, Marius Liviu Moise, Octavian Neagoe, Ioana Mihaela Citu, Bogdan Feciche, Florin Gorun, Dragos Erdelean, Adrian Ratiu, Cosmin Citu

**Affiliations:** 1 Department of Obstetrics and Gynecology, “Victor Babes” University of Medicine and Pharmacy Timisoara, Eftimie Murgu Square 2, 300041 Timisoara, Romania; 2 Doctoral School, “Victor Babes” University of Medicine and Pharmacy Timisoara, Eftimie Murgu Square 2, 300041 Timisoara, Romania; 3 Department of Anatomy and Embryology, “Victor Babes” University of Medicine and Pharmacy Timisoara, Eftimie Murgu Square 2, 300041 Timisoara, Romania; 4 Department of Infectious Diseases, “Victor Babes” University of Medicine and Pharmacy Timisoara, Eftimie Murgu Square 2, 300041 Timisoara, Romania; 5 Department of Family Medicine, “Victor Babes” University of Medicine and Pharmacy Timisoara, Eftimie Murgu Square 2, 300041 Timisoara, Romania; 6 Department of Radiology, “Premiere” Hospital—“Regina Maria,” Calea Aradului 113, 300643 Timisoara, Romania; 7 First Department of Surgery, Second Discipline of Surgical Semiology, “Victor Babes” University of Medicine and Pharmacy Timisoara, Eftimie Murgu Square 2, 300041 Timisoara, Romania; 8 Department of Internal Medicine I, “Victor Babes” the University of Medicine and Pharmacy Timisoara, Eftimie Murgu Square 2, 300041 Timisoara, Romania; 9 Department of Urology, Satu-Mare County Emergency Hospital, Strada Ravensburg 2, 440192 Satu-Mare, Romania

**Keywords:** SARS-CoV-2, COVID-19, pregnancy infections, premature birth, prematurity

## Abstract

Studies observed that women infected with SARS-CoV-2 during pregnancy had a higher risk of preterm birth. Although it is likely that COVID-19 during the late trimester of pregnancy can trigger premature birth, prematurity remains a concern, and it is vital to study additional clinical and biological patient factors that are highly associated with this negative pregnancy outcome and allow for better management based on the existing predictors. In order to achieve this goal, the current study retrospectively recruited 428 pregnant patients that were separated into three study groups using a 1:2:4 matching ratio and a nearest-neighbor matching method. Sixty-one pregnant patients had a history of COVID-19 during pregnancy and gave birth prematurely; 124 pregnant patient controls had COVID-19 and gave birth full-term, while the second control group of 243 pregnant patients had a premature birth but no history of COVID-19. It was observed that a symptomatic SARS-CoV-2 infection during the third trimester was significantly more likely to be associated with premature birth. Even though the rate of ICU admission was higher in these cases, the mortality rate did not change significantly in the COVID-19 groups. However, SARS-CoV-2 infection alone did not show statistical significance in determining a premature birth (β = 1.09, CI = 0.94–1.15, *p*-value = 0.067). Maternal anemia was the strongest predictor for prematurity in association with SARS-CoV-2 infection (β = 3.65, CI = 1.46–5.39, *p*-value < 0.001), followed by elevated CRP (β = 2.11, CI = 1.20–3.06, *p*-value < 0.001), and respectively IL-6 (β = 1.92, CI = 1.20–2.47, *p*-value = 0.001. SARS-CoV-2 infection is associated with an increased risk of preterm birth, as shown by our data. If SARS-CoV-2 infection arises during the third trimester, it is recommended that these patients be hospitalized for surveillance of clinical evolution and biological parameters, such as anemia and high inflammatory markers, which have a multiplicative influence on the pregnancy result.

## 1. Introduction

Pregnancy status does not seem to increase the likelihood of COVID-19, and the majority of pregnant patients with SARS-CoV-2 infection tend to recover before the moment of giving birth [1,2]. In pregnant women infected with SARS-CoV-2, the rates of mild, moderate, and severe disease are comparable to those in the general population [3,4,5], although some data suggests that pregnant women diagnosed with COVID-19 had a higher rate of more severe complications than the general population, due to pregnancy-specific comorbidities overlapping the acute SARS-CoV-2 infection [6,7,8]. It is hypothesized that viral infections during the pregnancy period may affect the moment of delivery, determining a premature birth, fetal health, and development, as well as increasing the risk for infertility [9,10,11,12]; therefore, a probable consequence of COVID-19 infection during pregnancy, a syndrome resembling preeclampsia has been reported during the second and third trimesters, increasing the fetal health risk as well as the likelihood of giving birth prematurely [13,14].

Similarly, SARS-CoV-2-infected pregnant women, especially those who develop pneumonia, seem to have a greater incidence of preterm birth and the necessity for cesarean delivery [15,16]. However, several elements of prenatal care among these higher-risk pregnancies with COVID-19, such as fetal monitoring, invasive prenatal diagnosis, the timing of delivery, and intrapartum monitoring, show conflicting evidence about the actual risks of SARS-CoV-2 infection during pregnancy [17]. Available information on pregnancy outcomes in SARS-CoV-2-infected women becomes more widely available, describing study participants who tested positive for COVID-19 when hospitalized for birth or pregnancy termination, although many have an unknown period of infection or no stratification for pregnancy groups [18,19].

Still, the majority of studies reported pregnancy outcomes during COVID-19 for the whole cohort and not by trimester of infection, few of them providing information about the pregnancy outcomes of women who were infected with SARS-CoV-2 during the early stages of pregnancy, the second trimester, and separately during the late stages of pregnancy [20,21]. Moreover, it is important to acknowledge that developed countries have successfully implemented the COVID-19 vaccination campaign among all population categories, including pregnant women, with a high safety standard [22,23]. Similarly, pregnant women in developed countries are more likely to have better medical care, self-care, and appropriate treatment during the pregnancy period, reducing the risks of complications [24,25,26]. However, there are still many countries with low vaccination coverage and higher rates of symptomatic SARS-CoV-2 infections, raising the concern over risks of prematurity and low birth weight.

Although some viruses may be transferred vertically to the fetus, there is minimal evidence that SARS-CoV-2 presents a significant risk of placental transmission [27]. During pregnancy, the placenta physically inhibits vertical transmission and has developed potent antimicrobial defenses. To date, it is uncertain whether the SARS-CoV-2 virus has found mechanisms to circumvent these defense mechanisms [28]. However, research on the consequences of SARS-CoV-2 infection during the first trimester of pregnancy reveals low miscarriage rates during the first trimester but higher proportions of preterm delivery if COVID-19 is discovered during the third trimester [29,30].

It was recently described that women infected with SARS-CoV-2 during their third trimester have an increased risk of preterm delivery compared to women without COVID-19 during pregnancy of the same gestational age [31]. There were no differences in pregnancy outcomes between infected and uninfected women throughout the first two trimesters of pregnancy, as well as other results highlighted that there were no differences in newborns that were small for gestational age and pregnancy loss [32]. Still, the risk for prematurity exists, being necessary to investigate other particular patients’ clinical and biological characteristics that are significantly correlated with giving birth prematurely. Therefore, the current study aimed to investigate the clinical and biological features of pregnant women with COVID-19 infection during the pregnancy period as risk factors for the onset of premature birth.

## 2. Materials and Methods

### 2.1. Study Ethics and Design

The study was performed by following a retrospective observational design that focused on pregnant women who contracted SARS-CoV-2 during pregnancy and were admitted or evaluated at the Obstetrics and Gynecology Clinic of the Timisoara Municipal Emergency Hospital between 1 March 2020, and 31 December 2021. Patient controls without a history of COVID-19 were screened from 1 January 2018 until 31 December 2021. The project included constructing the cohort and collecting data for three separate study groups. The study was approved by the Ethics Committee of the “Victor Babes” University of Medicine and Pharmacy and by the Ethics Committee of the Timisoara Municipal Hospital, with the approval number E-2814 from 19 May 2022.

### 2.2. Inclusion and Exclusion Circumstances

All adult pregnant women with a documented history of SARS-CoV-2 infection during pregnancy were considered for study inclusion. Pregnancy was confirmed by an hCG blood test, while the diagnosis of COVID-19 was considered after a positive RT-PCR, according to guidelines [33,34]. Patients with a confirmed SARS-CoV-2 infection before the moment of a confirmed pregnancy test were excluded from the study. COVID-19 vaccination status was also considered for inclusion since recent findings suggest similar safety levels for pregnant women as for the general population [35,36,37]. In order to compare the relevant outcomes, two control groups were designed, one group of pregnant women with COVID-19 during pregnancy and a full-term birth and a second control group of pregnant women without a history of SARS-CoV-2 infection during pregnancy who gave birth prematurely. The patient selection was performed using a 1:2:4 matching ratio by age and comorbidities, known as independent risk factors for premature birth, to avoid the effects of confounding factors. A nearest-neighbor matching method was used to select the patients. Our researchers recruited a total of 428 eligible patients during the study period, where 61 were cases of prematurity among COVID-19 mothers, 124 cases of full-term births among COVID-19 mothers, and a larger control group of 243 cases of prematurity among pregnant women without SARS-CoV-2 infection during pregnancy. Pregnancies without SARS-CoV-2 infection during the COVID-19 pandemic were identified when the patients presented for regular pregnancy checks with a negative PCR test performed within 72 h before the visit. Exclusion criteria for the research include refusal to provide personal medical data, as identified in the paper records, underage pregnancy, and difficulty understanding informed consent.

### 2.3. Study Variables and Definitions

The relevant data and variables considered for assessment in the current study were extracted from medical records, and comprised the following: (I) patients’ demographics—general characteristics (age, pre-pregnancy BMI), obstetrical characteristics (previous number of pregnancies and number of births, history of twin birth, history of induced abortion), comorbidities, COVID-19 vaccination status, and smoking status; (II) patient outcomes during previous and current pregnancy—pregnancy complications, trimester of SARS-CoV-2 infection, and the presence of symptomatic COVID-19; (III) neonatal outcomes—the presence of anemia, puerperal infection, neonatal respiratory distress syndrome (NRDS), meconium aspiration, small for gestational age status, sepsis, intensive care unit (ICU) admissions, and mortality, birth weight, severity of prematurity, and APGAR score; (IV) biological parameters—red blood cell count (RBC), PLT (platelets), WBC (white blood cell count), lymphocyte count, hemoglobin levels, hematocrit, creatinine, blood urea nitrogen (BUN), glomerular filtration rate (GFR), fasting glucose levels, alanine aminotransferase (ALT), aspartate aminotransferase (AST), ferritin, lactate dehydrogenase (LDH), procalcitonin, c-reactive protein (CRP), interleukin 6 (IL-6), erythrocyte sedimentation rate (ESR), fibrinogen, and D-dimers. Anemia was defined by below-the-normal range values of RBC or hemoglobin levels below 11 g/dL, according to World Health Organization (WHO) guidelines [38].

### 2.4. Statistical Analysis

The statistical analysis was performed with IBM SPSS v.27 (SPSS. Inc., Chicago, IL, USA), while the significance threshold was set for an alpha value of 0.05. The absolute and relative frequencies of categorical variables were computed and compared using the Chi-square and Fisher’s tests. The comparison of mean rank differences among nonparametric variables was performed with the Kruskal-Wallis test. Continuous variables that followed a normal distribution were compared by mean and standard deviation with the ANOVA test (analysis of variance). A Kaplan-Meier curve was plotted for the probability of prematurity, while the Cox regression identified the hazard ratio for prematurity. The multiple linear regression analysis identified the significant determinants for premature birth.

## 3. Results

### 3.1. Background Analysis

During the selection process, a total of 428 pregnant women were included in the final analysis, after excluding 179 records, according to the study criteria. There were no incomplete records from the COVID-19 prematurity group, 31 incomplete records in the COVID-19 no prematurity group, respectively 65 incomplete patient records among pregnant patients without a history of SARS-CoV-2 infection which gave birth prematurely. Other reasons for exclusion were the lack of signatures in the paper records allowing for personal data to be used in research. There were two patients who did not consent in the first group, 29 in the second group, and 52 in the third group. After the selection process, we identified 61 eligible pregnant women with a history of SARS-CoV-2 infection during the last pregnancy that resulted in preterm birth. From this starting point, a matched group on a 2:1 ratio was selected among pregnant women positive for COVID-19 during their pregnancy who gave birth at full term. A second control group consisting of women who gave birth prematurely without COVID-19 infection was matched 4:1 to the study group.

The demographics analysis presented in Table 1 described no statistically significant differences between the age, body mass index, number of previous pregnancies and births, as well as the concerning comorbidities. The average age of all pregnant women included in the study was approximately 30 years old, while the BMI suggested that the majority of patients were normally weighted according to their gestational age. The most common comorbid condition was high blood pressure of primary origin, followed by UTIs and depression, without significant differences in proportions between the three groups. Regarding the COVID-19 vaccination status, there were approximately 7% of patients with at least one dose, without any important differences between study groups.

**Figure 1:**
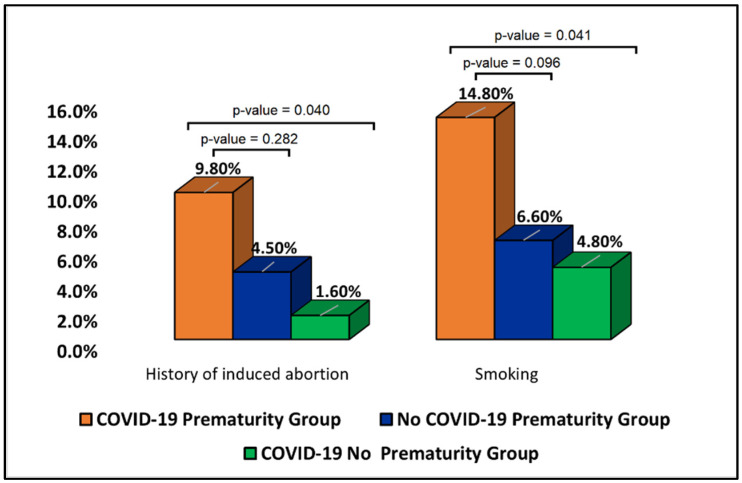
Proportions of patients with a history of induced abortion and smoking.

Although the history of pregnancy loss did not differ significantly between groups, it was observed that the history of induced abortion was significantly higher in the COVID-19 prematurity group compared with the no prematurity group. As seen in Figure 1, 9.8% of women in the COVID-19 prematurity group had a history of induced abortion, compared with 1.6% in the no prematurity group of COVID-19 patients (*p*-value = 0.040). However, the difference was not statistically significant when compared to the no-COVID-19 group of pregnant women who gave birth prematurely. Similarly, pregnant women with SARS-CoV-2 infection who gave birth prematurely were smoking in a higher proportion, compared with the no prematurity group (14.8% smokers vs. 4.8%, *p*-value = 0.041). However, the significance was lower when compared with the control group without COVID-19 (14.8% vs. 6.6%, *p*-value = 0.096), likely to indicate the impact of smoking as a confounding factor for giving birth prematurely.

### 3.2. Patient Outcomes

**Figure 2:**
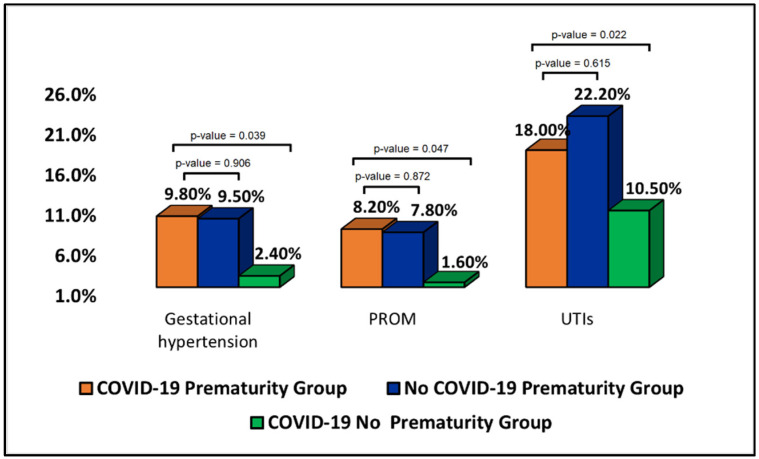
Proportions of patients with gestational hypertension, premature rupture of membranes, and urinary tract infections during previous pregnancies.

The patient’s medical history and current pregnancy outcomes, presented in Table 2, identified that the pregnant women with COVID-19 who gave birth prematurely had similar trends with the control group of patients with preterm births without COVID-19 infection. Thus, it was observed that gestational hypertension was a complication more frequently affecting the two prematurity groups (9.8% vs. 9.5% vs. 2.4% in the COVID-19 no prematurity group, *p*-value = 0.039). Similarly, PROM and UTIs were significantly more likely to be found in the group of patients who gave birth prematurely, regardless of the COVID-19 status during pregnancy, as presented in Figure 2. Among current pregnancy complications, anemia was statistically significantly more prevalent among the two prematurity groups compared to the control group with full-term births. There was no significant difference between the COVID-19 prematurity group and the control group of no COVID-19 mothers who gave birth prematurely (24.6% vs. 24.3%, *p*-value = 0.903).

Another important finding was that patients who gave birth prematurely had significantly more SARS-CoV-2 infections during the late stages of pregnancy compared with the control group (47.5% of patients with COVID-19 in the d-trimester, compared with 32.3% in the control group with full-term births and COVID-19, *p*-value = 0.005). Moreover, it was observed that the same patients in the COVID-19 prematurity group were significantly more likely to have a symptomatic infection (76.4% vs. 47.6%, *p*-value = 0.021).

### 3.3. Neonatal Outcomes

**Figure 3:**
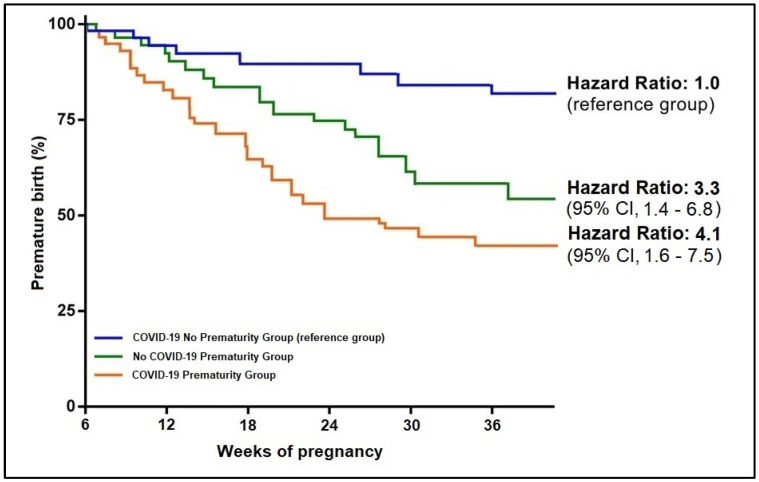
Kaplan-Meier probability analysis of premature birth by study group.

The neonatal outcomes presented in Table 3 identified no ICU admission in the group of 124 neonates born at full term. However, in the two groups of prematurity, the ICU admission rate was 9.8% among children born from a mother with SARS-CoV-2 infection during pregnancy, respectively 7.8% ICU admissions among those who were born prematurely from a mother without COVID-19. As expected, the birth weight distribution was statistically significantly different among the study groups, with a proportion of 19.7% of neonates born with an average weight between 1500 and 2500 g, compared to 4.8% in the COVID-19 no prematurity group and respectively 19.3% in the control group without SARS-CoV-2 infection (*p*-value = 0.001). Consequently, to a higher proportion of neonates with low birth weight, the APGAR score was significantly lower in the two prematurity groups, with the highest proportion of neonates with a score equal to or lower than six from the group of SARS-CoV-2 infected mothers (*p*-value < 0.001). Although there were four neonates with a positive PCR test for SARS-CoV-2, they presented no signs and symptoms of infection. The Kaplan-Meier analysis presented in Figure 3 identified a hazard ratio for prematurity of 3.3 among patients without COVID-19 during pregnancy. The risk increased to 4.1 among pregnant patients with SARS-CoV-2 infection during the pregnancy period.

### 3.4. Laboratory Analysis

In order to investigate the potential predisposing biological changes for premature birth among pregnant women that were infected with SARS-CoV-2, a parallel comparison of biological parameters at admission and discharge was comprised in Table 4. Among the few important findings, it was observed that admission serum markers were statistically significantly more altered than parameters at discharge. During admission for COVID-19, the pregnant women who later gave birth prematurely had significantly lower hemoglobin (27.9% patients with low hemoglobin levels, compared to 15.3% in the no prematurity group, *p*-value = 0.042). The median value of hemoglobin in the prematurity group was 11.4 g/dL, compared with a median hemoglobin level of 12.6 g/dL (*p*-value = 0.039). Other significant differences were observed in inflammatory markers CRP (22.1 mg/L vs. 17.4 mg/L, *p*-value = 0.020), IL-6 (21.5 pg/mL vs. 17.9 pg/mL, *p*-value = 0.009), and respectively procalcitonin levels (1.1 ng/mL vs. 0.8 ng/mL, *p*-value = 0.044). A total of 12 patients (19.7%) from the COVID-19 prematurity group had elevated procalcitonin levels, compared to only 8.9% in the control group (*p*-value = 0.036).

At discharge, it was observed that the majority of analyzed biological parameters normalized, except the fasting glucose levels, which increased from 8.2% to 18.0% abnormal samples, as well as lower creatinine levels (from 4.9% to 9.8%), and respectively more altered liver markers, that were elevated in 24.6% of pregnant women with COVID-19 who gave birth prematurely, compared to only 11.5% at admission. It is likely that these findings are attributed to the treatment protocols for admitted COVID-19 patients.

Table 5 describes the parallel analysis of biological parameters at birth between the three study groups. Among the significant findings, it was observed that the white blood cell count was significantly more elevated in the COVID-19 prematurity group, likely due to the late pregnancy infection with SARS-CoV-2 (26.2% elevated samples, vs. 11.3% in the COVID-19 no prematurity group, respectively 15.6% in the no COVID-19 prematurity group, *p*-value = 0.042). Other significant findings were the presence of anemia more frequently in the COVID-19 prematurity group in 21.3% of patients, compared to the no COVID-19 prematurity group (11.9%, *p*-value = 0.037). Lastly, the CRP levels were more elevated among pregnant women with SARS-CoV-2 infection who gave birth prematurely, compared with the control group of premature births without a history of COVID-19 (16.4% vs. 7.4%, *p*-value = 0.024).

A multiple linear regression model was constructed in Table 6 to observe the predictors for premature birth in COVID-19 pregnant women since SARS-CoV-2 infection alone did not show statistical significance in determining a premature birth (β = 1.09, CI = 0.94–1.15, *p*-value = 0.067). The significant predictors from clinical findings were smoking, gestational hypertension, PROM, UTIs, as well as the SARS-CoV-2 infection during the third trimester of pregnancy (β = 1.55, CI = 1.38–2.93, *p*-value = 0.014), and a having a symptomatic infection (β = 1.23, CI = 1.09–1.38–2.21, *p*-value = 0.036).

**Figure 4:**
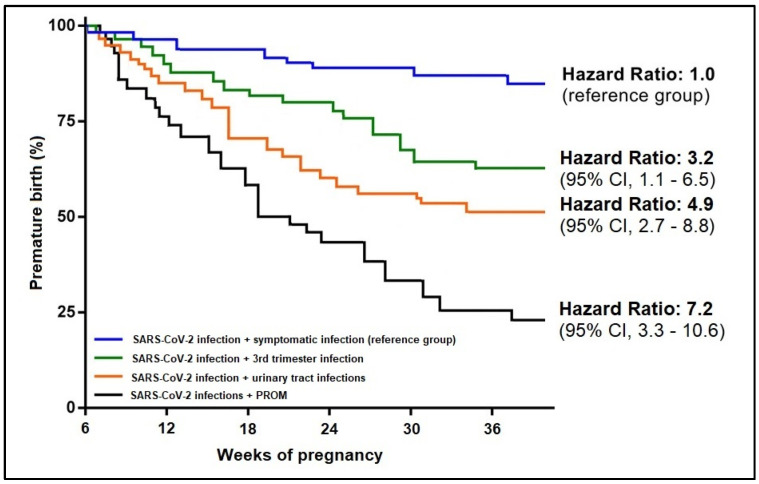
Kaplan-Meier probability analysis of premature birth by clinical predictors.

**Figure 5:**
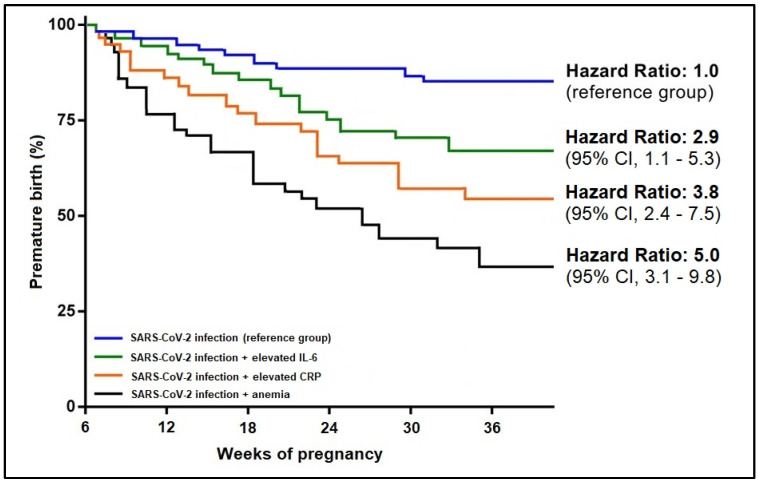
Kaplan-Meier probability analysis of premature birth by biological predictors.

In addition to the significant clinical findings, some biological parameters were found to have a significant influence on the onset of premature birth in association with the SARS-CoV-2 infection. Anemia was the strongest predictor in association with COVID-19 (β = 3.65, CI = 1.46–5.39, *p*-value < 0.001), followed by elevated CRP (β = 2.11, CI = 1.20–3.06, *p*-value < 0.001), and respectively IL-6 (β = 1.92, CI = 1.20–2.47, *p*-value = 0.001). In the risk analysis for premature birth among SARS-CoV-2 infected pregnant women, the Kaplan-Meier probability curves presented in Figure 4 and Figure 5 showed that SARS-CoV-2 infection associated with PROM had a 7.2 times higher likelihood of premature birth, as compared to the reference group of symptomatic SARS-CoV-2 infections. From the biological parameter’s standpoint, COVID-19 associated with anemia had five times the higher likelihood that SARS-CoV-2 infection alone (reference group), followed by the association with elevated CRP (HR = 3.8) and elevated IL-6 (HR = 2.9).

## 4. Discussion

### 4.1. Important Findings

The current study identified various clinical and biological features of pregnant women with SARS-CoV-2 infection during the pregnancy period as risk factors for premature birth. The primary merit of the study is the analysis of pregnant women patients in comparison with two control groups in order to validate the significant findings. Among the most important observations, it is to be mentioned that SARS-CoV-2 infection alone did not prove to be a significant independent risk factor for the onset of premature birth. However, several regression models identified significant predictors for prematurity in association with the COVID-19 diagnosis during the pregnancy period. In addition to the already known significant risk factors for prematurity (smoking, gestational hypertension, PROM, and UTIs), it was observed that a symptomatic SARS-CoV-2 infection during the third trimester of pregnancy had a significant impact on the moment of birth. Among biological findings, anemia, elevated CRP, and IL-6 were also significant predictors for prematurity in association with SARS-CoV-2 infection during pregnancy.

As described by another large-scale study on SARS-CoV-2 implications during pregnancy, pregnant women with a positive COVID-19 diagnosis during the third trimester of pregnancy had an enhanced risk of premature birth, although pregnant women infected early during pregnancy had similar risks to the general population [39]. These findings are consistent with a number of earlier studies about SARS-CoV-2 infections in late pregnancy or at the time of birth, although the statistical power was insufficient to find any unfavorable pregnancy outcomes since during the early stages of the pandemic the number of pregnant patients infected with SARS-CoV-2 was low [40,41]. It is important to mention the observed elevated incidence of premature birth from previous research that indicated it was most pronounced among pregnant patients infected during the third trimester and exhibiting a symptomatic infection. However, causality was not determined, and these associations were limited to a small number of observations [42,43]. It was later described by a meta-analysis that 98% of infections occurred during the third trimester when prematurity was involved, although it was suggested that doctors were more likely to induce labor in women infected with SARS-CoV-2 to prevent complications, biasing the natural onset of preterm birth [44,45]. Over fifty percent of infections throughout the first and second trimesters compared to earlier research might explain the reduced risk projections derived by our study.

Although we observed higher rates of prematurity among SARS-CoV-2 infected women during their pregnancy, there was no difference in the prevalence of diagnoses of small for gestational age as compared with the control groups of COVID-19 mothers who gave birth full term and those who gave birth prematurely but in the absence of SARS-CoV-2 infection during pregnancy (16.4% vs. 7.3% vs. 15.6%), which is consistent with the existing literature and suggests that COVID-19 during pregnancy is unlikely to be associated with intrauterine growth limitation [46,47].

Regarding the biological findings of the current study, fasting glucose was observed to be significantly more elevated at discharge after SARS-CoV-2 infection, likely due to corticosteroid use in COVID-19 patients, according to existing protocols. Corticosteroids stand out among the multitude of medications used to treat COVID-19 infection because they reduce mortality among hospitalized severely to critically sick patients. Seeing this as a glimpse of optimism, medical professionals have begun employing these medications more often than ever before in clinical practice [48]. The fear of short-term death has trumped the worry about the long-term effects of steroid usage, while the convenience of availability, cheap cost, and apparent short-term therapeutic benefit have led to the irresponsible use of steroids, including self-medication, even in moderate COVID-19 patients. In COVID-19 patients, the administration of corticosteroids has increased the incidence of elevated serum glucose levels and the acute consequences of diabetic ketoacidosis that can negatively influence pregnancy and determine future complications among newborns [49,50].

In a recent retrospective investigation, the authors determined that elevated ferritin and C-reactive protein blood levels were related to a poor prognosis and death in unvaccinated pregnant women infected with SARS-CoV-2 [51]. Moreover, a cross-sectional study evaluating the clinical course of maternal mortality cases due to severe forms of COVID-19 found a higher frequency of leukocytosis with elevated neutrophils to lymphocytes ratio, low platelet count, and elevated levels of inflammatory markers such as CRP, serum ferritin, LDH, D-dimers, and serum fibrinogen in their cohort of unvaccinated pregnant patients. These findings are partially in accordance with the current study, as only about 6% of all studied patients were vaccinated against COVID-19. Still, anemia, CRP, and IL-6 were significantly associated with prematurity in SARS-CoV-2-infected pregnant women in our study.

### 4.2. Study Limitations and Future Perspectives

One limitation faced by our study is the retrospective design. Therefore, the absence of COVID-19 during pregnancy was validated only if the patient presented to the clinic for the usual pregnancy checks by bringing a negative PCR test performed in the last 72 h prior to the visit instead of a more reliable method by a negative serology test. Although the study groups were matched by age and comorbid conditions that are known risk factors for prematurity, there are still several biasing factors that couldn’t be eliminated in this study, such as smoking and the history of gestational hypertension, to avoid reducing the sample size to insignificant levels. Even though patients were case-matched when included in the current study, the disease severity was not taken into consideration. Therefore, different results might occur due to these factors.

Further prospective studies are needed to identify the long-term effects of SARS-CoV-2 infection during pregnancy, both on pregnant women and the child. The long-term effects of children that acquire SARS-CoV-2 infection during or after delivery should also be explored further. There are still unexplored biological markers that might better predict the outcome of the pregnancy in SARS-CoV-2-infected pregnant women and allow for better management. It could further be explored the weeks of pregnancy that are most likely to be influenced by COVID-19 and determine a premature birth.

## 5. Conclusions

Our findings support the idea that SARS-CoV-2-positive pregnant women do not expose the fetus to an additional risk of intrauterine growth restriction or significant complications. Although not posing a concern to all pregnant women with COVID-19, preterm delivery can be expected if the mother develops a symptomatic SARS-CoV-2 infection in the later stages of pregnancy. Women in their third trimester should maintain social distance and use face masks to reduce the chance of adverse pregnancy outcomes, such as premature birth. If, however, SARS-CoV-2 infection occurs during the third trimester, it is advisable for these patients to be admitted to the hospital for observation of clinical evolution and biological parameters such as anemia and elevated inflammatory markers with an additive effect on the outcome of the pregnancy.

## Figures and Tables

**Table 1:** Demographics of pregnant women included in the study.

Variables *	COVID-19 Prematurity Group (*n* = 61)	COVID-19 No Prematurity Group (*n* = 124)	No COVID-19 Prematurity Group (*n* = 243)	*p*-Value
General characteristics				
Age (years), mean ± SD	30.1 ± 4.8	29.6 ± 4.6	28.9 ± 4.9	0.147
Pre-pregnancy BMI, mean ± SD	22.5 ± 2.9	22.7 ± 2.5	22.1 ± 2.8	0.122
Previous pregnancies				0.920
1	38 (62.3%)	76 (61.3%)	150 (61.7%)	
2	14 (23.0%)	29 (23.4%)	63 (25.9%)	
≥3	9 (14.8%)	19 (15.3%)	30 (12.3%)	
Number of births				0.348
0	4 (6.6%)	8 (6.5%)	28 (11.5%)	
1	41 (67.2%)	90 (72.6%)	155 (63.8%)	
≥2	16 (26.2%)	26 (21.0%)	60 (24.7%)	
History of twin birth	1 (1.6%)	1 (0.8%)	3 (1.2%)	0.874
Comorbidities **				
Diabetes Mellitus	2 (3.3%)	5 (4.0%)	10 (4.1%)	0.955
Asthma	4 (6.6%)	8 (6.5%)	14 (5.8%)	0.952
Coagulation disorders	2 (3.3%)	3 (2.4%)	7 (2.9%)	0.940
High blood pressure	9 (11.8%)	11 (8.9%)	28 (11.5%)	0.478
Thyroid disorders	1 (1.6%)	2 (1.6%)	5 (2.1%)	0.946
UTI	5 (8.2%)	10 (8.1%)	19 (7.8%)	0.993
Depression	6 (9.8%)	14 (11.3%)	23 (9.5%)	0.858
Others	3 (4.9%)	5 (4.0%)	12 (4.9%)	0.922
History of pregnancy loss				0.112
Yes	7 (11.5%)	6 (4.8%)	13 (5.3%)	
No	54 (88.5%)	118 (95.2%)	230 (94.7%)	
History of induced abortion				0.040
Yes	6 (9.8%)	2 (1.6%)	11 (4.5%)	
No	53 (86.9%)	119 (96.0%)	228 (94.7%)	
COVID-19 vaccination status				0.815
Yes	4 (6.6%)	7 (5.6%)	18 (7.4%)	
No	57 (93.4%)	117 (94.4%)	58 (92.6%)	
COVID-19 vaccine doses				0.824
1	1 (25.0%)	3 (42.9%)	6 (33.3%)	
≥2	3 (75.0%)	4 (57.1%)	12 (66.7%)	
Smoking status				0.041
Yes	9 (14.8%)	6 (4.8%)	16 (6.6%)	
No	52 (85.2%)	118 (95.2%)	227 (93.4%)	

* Data is presented as *n* (%) unless specified differently; ** Identified before pregnancy; UTI—Urinary Tract Infections; BMI—Body Mass Index; BMI—Body.

**Table 2:** Patient outcomes.

Variables *	COVID-19 Prematurity Group (*n* = 61)	COVID-19 No Prematurity Group (*n* = 124)	No COVID-19 Prematurity Group (*n* = 243)	*p*-Value
Complications during previous pregnancies				
Pregnancy-associated DM	3 (4.9%)	7 (5.6%)	11 (4.5%)	0.895
Gestational hypertension	6 (9.8%)	3 (2.4%)	23 (9.5%)	0.039
Preeclampsia	2 (3.3%)	1 (0.8%)	8 (3.3%)	0.338
Abnormal placental implantation	4 (6.6%)	3 (2.4%)	13 (5.3%)	0.341
PROM	5 (8.2%)	2 (1.6%)	19 (7.8%)	0.047
UTIs	11 (18.0%)	13 (10.5%)	54 (22.2%)	0.022
Cesarean delivery	14 (23.0%)	22 (17.7%)	55 (22.6%)	0.523
Oligohydramnios	0 (0.0%)	1 (0.8%)	6 (2.5%)	0.273
Polyhydramnios	2 (3.3%)	2 (1.6%)	11 (4.5%)	0.354
Anemia	12 (19.7%)	18 (14.5%)	52 (21.4%)	0.283
Endometritis	2 (3.3%)	0 (0.0%)	7 (2.9%)	0.150
Trimester of SARS-CoV-2 infection				0.005
1st trimester	9 (14.8%)	47 (37.9%)	–	
2nd trimester	23 (37.7%)	37 (29.8%)	–	
3rd trimester	29 (47.5%)	40 (32.3%)	–	
Symptomatic SARS-CoV-2 infection				0.021
Yes	40 (76.4%)	59 (47.6%)	–	
No	21 (23.6%)	65 (52.4%)	–	
Current pregnancy complications				
Pregnancy-associated DM	4 (6.6%)	6 (4.8%)	18 (7.4%)	0.642
Gestational hypertension	7 (9.8%)	1 (0.8%)	26 (10.7%)	0.002
Preeclampsia	1 (1.6%)	2 (1.6%)	5 (2.1%)	0.946
Abnormal placental implantation	2 (3.3%)	3 (2.4%)	13 (5.3%)	0.341
PROM	6 (9.8%)	2 (1.6%)	17 (7.0%)	0.040
UTIs	13 (21.3%)	14 (11.3%)	58 (23.9%)	0.016
Cesarean delivery	16 (26.2%)	25 (20.2%)	61 (25.1%)	0.514
Oligohydramnios	2 (3.3%)	0 (0.0%)	6 (2.5%)	0.173
Polyhydramnios	3 (4.9%)	1 (0.8%)	7 (2.9%)	0.219
Anemia	15 (24.6%)	16 (12.9%)	59 (24.3%)	0.031
Endometritis	4 (6.6%)	4 (3.2%)	12 (4.9%)	0.574

* Data is presented as *n* (%) unless specified differently; UTI—Urinary Tract Infections; DM—Diabetes Mellitus; PROM—Premature Rupture of Membranes.

**Table 3:** Neonatal outcomes.

Variables *	COVID-19 Prematurity Group (*n* = 61)	COVID-19 No Prematurity Group (*n* = 124)	No COVID-19 Prematurity Group (*n* = 243)	*p*-Value
Neonatal Outcomes				
Anemia	9 (14.8%)	16 (12.9%)	40 (16.5%)	0.664
Puerperal infection	3 (4.9%)	3 (2.4%)	11 (4.5%)	0.570
NRDS	3 (4.9%)	1 (0.8%)	12 (4.9%)	0.124
Meconium aspiration	5 (4.0%)	8 (6.5%)	22 (9.1%)	0.690
Small for gestational age	10 (16.4%)	9 (7.3%)	38 (15.6%)	0.061
Sepsis	4 (6.6%)	1 (0.8%)	14 (5.8%)	0.063
ICU admission	6 (9.8%)	0 (0.0%)	19 (7.8%)	0.003
Mortality	1 (1.6%)	0 (0.0%)	5 (2.1%)	0.280
SARS-CoV-2 infection	2 (3.3%)	2 (1.6%)	–	0.463
Birth weight				0.001
<1500 g	1 (1.6%)	0 (0.0%)	5 (2.1%)	
1500–2500 g	12 (19.7%)	6 (4.8%)	47 (19.3%)	
>2500 g	48 (82.7%)	118 (93.4%)	191 (94.6%)	
Severity of prematurity				0.670
Moderate to late preterm (32–37 weeks)	42 (68.9%)	–	177 (72.8%)	
Very preterm (28–32 weeks)	16 (26.2%)	–	59 (24.3%)	
Extremely preterm (<28 weeks)	3 (4.9%)	–	7 (2.9%)	
APGAR score				<0.001
≥9	7 (11.5%)	78 (62.9%)	32 (13.2%)	
7–8	40 (65.6%)	34 (27.4%)	172 (70.8%)	
≤6	14 (23.0%)	12 (9.7%)	39 (16.0%)	

* Data is presented as *n* (%) unless specified differently; ICU—Intensive Care Unit; APGAR—Appearance, Pulse, Grimace, Activity, and Respiration; NRDS—Neonatal Respiratory Distress Syndrome.

**Table 4:** Laboratory analysis of pregnant women at admission and discharge for SARS-CoV-2 infection.

Variables *	Normal Range	At Admission		*p*-Value	Before Discharge		*p*-Value
		COVID-19 Prematurity Group (*n* = 61)	COVID-19 No Prematurity Group (*n* = 124)		COVID-19 Prematurity Group (*n* = 61)	COVID-19 No Prematurity Group (*n* = 124)	
RBC (millions/mm^3^)	4.35–5.65	16 (26.2%)	22 (17.7%)	0.179	13 (21.3%)	20 (16.1%)	0.386
PLT (thousands/mm^3^)	150–450	14 (23.0%)	19 (15.3%)	0.202	12 (19.7%)	16 (12.9%)	0.227
WBC (thousands/mm^3^)	4.5–11.0	38 (62.3%)	76 (61.3%)	0.894	22 (36.1%)	51 (41.1%)	0.507
Lymphocytes (thousands/mm^3^)	1.0–4.8	40 (65.6%)	65 (52.4%)	0.089	23 (37.7%)	31 (25.0%)	0.079
Hb (g/dL)	11.0–15.0	17 (27.9%)	19 (15.3%)	0.042	14 (23.0%)	15 (12.1%)	0.056
Hematocrit (%)	30–37	13 (21.3%)	14 (11.3%)	0.069	10 (16.4%)	11 (8.9%)	0.129
Creatinine (µmol/L)	0.4–08	3 (4.9%)	3 (2.4%)	0.367	6 (9.8%)	6 (4.8%)	0.194
BUN (mmol/L)	2.1–8.5	4 (6.6%)	8 (6.5%)	0.978	7 (11.5%)	5 (4.0%)	0.053
GFR	>60	6 (9.8%)	5 (4.0%)	0.116	6 (9.8%)	7 (5.6%)	0.294
Fasting glucose (mg/dL)	<95	5 (8.2%)	10 (8.1%)	0.975	11 (18.0%)	14 (11.3%)	0.207
ALT (U/L)	7–35	7 (11.5%)	6 (4.8%)	0.096	15 (24.6%)	19 (15.3%)	0.126
AST (U/L)	10–40	8 (13.1%)	14 (11.3%)	0.718	12 (19.7%)	14 (11.3%)	0.123
Ferritin (ng/mL)	15–300	23 (37.7%)	31 (25.0%)	0.073	17 (27.9%)	26 (21.0%)	0.296
LDH (U/L)	100–300	9 (14.8%)	16 (12.9%)	0.729	6 (9.8%)	5 (4.0%)	0.116
Procalcitonin (ng/mL)	<0.5	12 (19.7%)	11 (8.9%)	0.036	9 (14.8%)	8 (6.5%)	0.066
CRP (mg/L)	0–10	29 (47.5%)	40 (32.3%)	0.043	11 (18.0%)	13 (10.5%)	0.150
IL-6 (pg/mL)	0–16	21 (34.4%)	23 (18.5%)	0.017	10 (16.4%)	12 (9.7%)	0.184
ESR (mm/h)	0–22	20 (32.8%)	29 (23.4%)	0.173	13 (21.3%)	19 (15.3%)	0.311
Fibrinogen (g/L)	2–4	24 (39.3%)	39 (31.5%)	0.286	13 (21.3%)	20 (16.1%)	0.386
D-dimers (ng/mL)	<250	18 (29.5%)	32 (25.8%)	0.594	16 (26.2%)	24 (19.4%)	0.285

* Data is presented as *n* (%) unless specified differently; The normal range was adjusted for pregnancy status; RBC—Red Blood Cells; PLT—Platelets; WBC—White Blood Cells; Hb—Hemoglobin; BUN—Blood Urea Nitrogen; GFR—Glomerular filtration Rate; CRP—C-reactive Protein; IL—Interleukin; ESR–Erythrocyte Sedimentation Rate; LDH—Lactate dehydrogenase.

**Table 5:** Parallel analysis of biological parameters at birth between the three study groups.

Variables *	Normal Range	COVID-19 Prematurity Group (*n* = 61)	COVID-19 No Prematurity Group (*n* = 124)	No COVID-19 Prematurity Group (*n* = 243)	*p*-Value
RBC (millions/mm^3^)	4.35–5.65	18 (29.5%)	26 (21.0%)	63 (25.9%)	0.664
PLT (thousands/mm^3^)	150–450	11 (18.0%)	16 (12.9%)	47 (19.3%)	0.300
WBC (thousands/mm^3^)	4.5–11.0	16 (26.2%)	14 (11.3%)	38 (15.6%)	0.042
Lymphocytes (thousands/mm^3^)	1.0–4.8	12 (19.7%)	20 (16.1%)	32 (13.2%)	0.404
Hb (g/dL)	11.0–15.0	13 (21.3%)	26 (21.0%)	29 (11.9%)	0.037
Hematocrit (%)	36–48	16 (26.2%)	24 (19.4%)	58 (23.9%)	0.497
Creatinine (µmol/L)	0.74–1.35	4 (6.6%)	6 (4.8%)	11 (4.5%)	0.805
BUN (mmol/L)	2.1–8.5	3 (4.9%)	4 (3.2%)	13 (5.3%)	0.656
GFR	>60	6 (9.8%)	14 (11.3%)	18 (7.4%)	0.446
Fasting glucose (mg/dL)	72–125	7 (11.5%)	20 (16.1%)	32 (13.2%)	0.629
ALT (U/L)	7–35	5 (8.2%)	10 (8.1%)	19 (7.8%)	0.993
AST (U/L)	10–40	4 (6.6%)	8 (6.5%)	14 (5.8%)	0.952
Ferritin (ng/mL)	15–300	14 (23.0%)	19 (15.3%)	63 (25.9%)	0.070
LDH (U/L)	100–300	6 (9.8%)	7 (5.6%)	27 (11.1%)	0.232
Procalcitonin (ng/mL)	<0.5	2 (3.3%)	1 (0.8%)	3 (1.2%)	0.382
CRP (mg/L)	0–10	11 (18.0%)	11 (8.9%)	18 (7.4%)	0.024
IL-6 (pg/mL)	0–16	8 (13.1%)	12 (9.7%)	22 (9.1%)	0.738
ESR (mm/h)	0–22	7 (11.5%)	19 (15.3%)	20 (8.2%)	0.113
Fibrinogen (g/L)	2–4	4 (6.6%)	7 (5.6%)	16 (6.6%)	0.937
D-dimer (ng/mL)	<250	8 (13.1%)	14 (11.3%)	15 (6.2%)	0.104

* Data is presented as *n* (%) unless specified differently; The normal range was adjusted for pregnancy status; RBC—Red Blood Cells; PLT—Platelets; WBC—White Blood Cells; Hb—Hemoglobin; BUN—Blood Urea Nitrogen; GFR—Glomerular filtration Rate; CRP—C-reactive Protein; IL—Interleukin; ESR–Erythrocyte Sedimentation Rate; LDH—Lactate dehydrogenase.

**Table 6:** Regression analysis for risk of premature birth in SARS-CoV-2 infected pregnant women.

	β for Premature Birth *	(95% CI of β)	Significance
SARS-CoV-2 infection (independent constant) **	1.09	0.94–1.15	0.067
Covariates (predictors)—clinical			
History of induced abortion	1.13	0.82–1.34	0.091
Smoking	2.07	1.25–2.84	0.002
Gestational hypertension	2.36	1.44–3.78	<0.001
PROM	1.94	1.13–3.69	<0.001
UTIs	1.82	1.27–3.22	<0.001
3rd Trimester of SARS-CoV-2 infection	1.55	1.38–2.93	0.014
Symptomatic SARS-CoV-2 infection	1.23	1.09–2.21	0.036
Covariates (predictors)—biological			
Anemia	3.65	1.46–5.39	<0.001
Elevated procalcitonin	1.09	0.91–1.43	0.063
Elevated CRP	2.11	1.34–3.06	<0.001
Elevated IL-6	1.92	1.20–2.47	0.001
Elevated WBC	1.13	0.98–1.42	0.051

* Dependent (response) variable; ** Estimated risk in univariate analysis; CI—Confidence Interval; CRP—C-reactive Protein; IL—Interleukin; WBC—White Blood Cells; UTI—Urinary Tract Infections; PROM—Premature Rupture of Membranes.

## Data Availability

The data presented in this study are available on request from the corresponding author.
